# Surface Analysis of Stainless Steel Electrodes Cleaned by Atmospheric Pressure Plasma

**DOI:** 10.3390/ma17143621

**Published:** 2024-07-22

**Authors:** Jia Zhang, Mengjia Dang, Cheng Luo, Yongshan Ba, Qingkai Li

**Affiliations:** School of Aerospace Science and Technology, Xidian University, Xi’an 710126, China; 22131214184@stu.xidian.edu.cn (M.D.); 21131110571@stu.xidian.edu.cn (C.L.); 23131213962@stu.xidian.edu.cn (Y.B.); 23131213886@stu.xidian.edu.cn (Q.L.)

**Keywords:** atmospheric pressure plasma, stainless steel electrode, carbon pollutants, wettability

## Abstract

The Z-pinch device is a critical component in inertial confinement fusion, where stainless steel electrodes must withstand high current densities of up to MA/cm^2^. Gases and difficult-to-remove impurities adhering to the electrode surfaces can ionize, significantly impacting the device’s electrical conductivity efficiency. In this paper, the surface of stainless steel electrodes was subjected to cleaning using a large-area plasma jet under atmospheric pressure. The wettability, chemical composition, and chemical state of the electrode surface were characterized using a water contact angle measuring instrument and X-ray photoelectron spectroscopy (XPS). The cleaning effect under different discharge parameters was systematically analyzed. The results revealed a significant reduction in the content of carbon pollutants on the surface of stainless steel electrodes, decreasing from 62.95% to a minimum of 37.68% after plasma cleaning. Moreover, the water contact angle decreased from 70.76° to a minimum of 29.31°, and the content of water molecules adsorbed on the surface decreased from 17.31% to a minimum of 5.9%. Based on the evolution process of micro-element content and chemical state on the surface of stainless steel electrode, the cleaning process of adhering substances on the surface by atmospheric pressure plasma was analyzed by the layered cleaning model for surface pollutants on stainless steel.

## 1. Introduction

The Z-pinch device serves as a critical component in inertial confinement fusion. The stainless steel electrode in the device needs to carry a large current density of MA/cm^2^ magnitude [1,2,3]. Under the condition of high current density, the adsorbed gases and attached substances on the surface of stainless steel electrodes will be ionized to form the electrode plasma due to bombardment by electrons and ions. The electrode plasmas significantly impact the current carrying capacity and efficiency of vacuum transmission lines [4]. The next generation of ultra-high-power Z-pinch devices demands higher efficiency in current transmission [5,6]. Consequently, it is essential to clean the electrode surfaces to remove adsorbed gases and stubborn impurities.

Plasma cleaning has emerged as a promising green technology in recent years, offering advantages such as operability, ease of control, pollution-free operation, and waste-free output [7,8,9,10,11,12]. This technology effectively removes pollutants like oxides, grease, and dust from metal surfaces, achieving thorough cleaning and enhancing surface purity [13,14,15]. During the cleaning process, high-energy particles within the plasma undergo chemical reactions with surface pollutants. These reactions convert both organic and inorganic pollutants into gases or other volatile substances. Additionally, the ions and electrons within the plasma possess elevated energy, enabling them to impact surface pollutants and dislodge them. This physical stripping mechanism aids in removing stubborn pollutants, such as grease and coatings. Furthermore, oxidation–reduction reactions readily occur in the plasma environment, facilitating the oxidation or reduction of certain pollutants and rendering them more amenable to removal. In general, plasma cleaning utilizes the high temperature and energy of the plasma environment, combining physical and chemical methods to remove pollutants from electrode surfaces, finding wide application in metal surface cleaning.

Low-pressure plasma cleaning technology emerged relatively early. In low-pressure environments, where gas density is reduced, the probability of electron collision decreases, resulting in less electron energy loss. Consequently, gas ionization is more probable under low pressure, generating a uniform plasma with higher density. This increases the probability of active particles combining with surface pollutants upon collision, thus facilitating its widespread application in metal surface cleaning [16,17]. However, the generation of low-pressure plasma necessitates a vacuum system, leading to high production costs. Furthermore, workpieces to be treated must be enclosed within vacuum chambers, limiting the size of treatable workpieces [18,19]. For larger-sized workpieces, atmospheric pressure plasma cleaning technology is preferable. Currently, atmospheric pressure plasma jets have been applied to varying degrees in cleaning metal surfaces, including aluminum [20,21], copper [22,23], chromium [24], stainless steel [25,26,27], and other metals. However, research on atmospheric pressure plasma cleaning technology faces challenges such as the small size of individual plasma jets and the large gap between arrayed plasma jets, which hinder the uniform cleaning of large-area samples.

This paper presented the design of a rotating array atmospheric pressure plasma jet generator, where the diameter of the plasma is greater than 60 mm, and its length exceeds 30 mm. Therefore, the large-sized objects have the potential to be cleaned, but the uniformity and effectiveness of plasma cleaning generated by this plasma jet are still unclear. This is an important aspect of the cleaning to consider as this will enable the use of this plasma jet to treat a large area.

Wettability is an important indicator of the cleanliness and activation level of surfaces. Therefore, this paper qualitatively analyzed the wettability of the electrode surface using water contact angle measurements. Additionally, the chemical composition and chemical states of the electrode surface were characterized using X-ray photoelectron spectroscopy (XPS), and the cleaning effects under different discharge powers and treatment times were analyzed. Based on the changes in activation level, chemical composition, and chemical states of the stainless steel electrode surface, the interaction process between the plasma jets and surface pollutants was analyzed. The mechanism by which plasma cleaning enhanced the wettability of stainless steel electrode surfaces was elucidated, providing support for the practical application of plasma cleaning technology.

## 2. Experimental

### 2.1. Plasma Cleaning Platform

**Figure 1:**
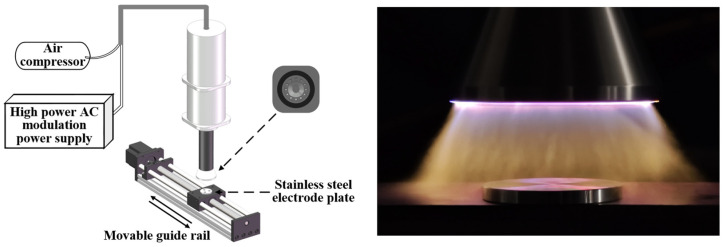
Atmospheric pressure plasma jet generation device and plasma jet.

The atmospheric pressure plasma jet generation device and the plasma jet produced for this experiment are depicted in Figure 1. The setup consists of the plasma jet spray gun, power supply unit, gas supply unit, and movable platform. The experimental power supply employed a high-power alternating current (AC) modulation power source, providing an output voltage of 30 kV and an output power ranging from 500 to 800 W. The output frequency was adjustable within the range of 25 to 35 kHz. The plasma jet generation device utilizes compressed air as the working gas with a pressure of 150 kPa. The plasma jet generation occurs through spark discharge, where plasma is ejected outward along with the high-pressure airflow, forming the plasma jet. The 304 stainless steel electrode samples are circular discs with a diameter of 30 mm and a thickness of 3 mm, possessing a surface roughness of 3.2 μm.

In our experiment, the jet outlet of the plasma generation device was composed of 12 circular nozzles each with a diameter of 5 mm, arranged in a circular pattern with a diameter of 45 mm. The nozzles can rotate at a very high speed, forming a ring-shaped plasma. At a distance of 20 mm to the jet outlet, the effective area of the ring-shaped plasma jet is approximately 8.9 cm^2^. Additionally, moving the sample at a certain rate allows for complete coverage cleaning of the sample surface.

**Figure 2:**
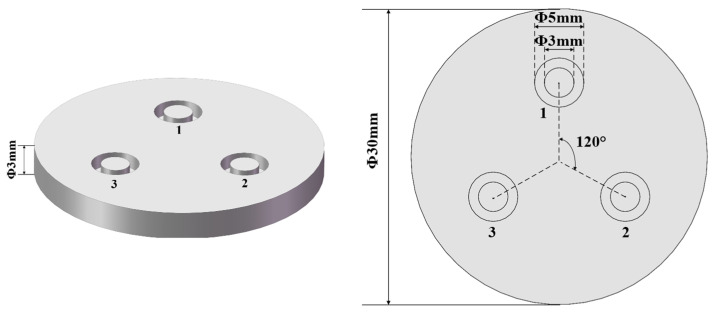
Three measuring points (1–3) on the surface of the stainless steel electrode.

Three measurement points are chosen on the surface of the stainless steel electrode to study the uniformity of plasma cleaning, as shown in Figure 2. The distance between the plasma jet nozzle and the electrode surface is set at 20 mm. Prior to plasma treatment, the samples are preliminarily cleaned using industrial alcohol with a concentration of 99%, followed by cleaning with the plasma jet. During the cleaning, the samples are placed on a movable platform, moving forwards and backwards over a distance of 12.5 cm at a speed of 1.25 cm/s. The discharge powers set in this experiment were 550 W and 750 W, with cleaning times of 0.5 min, 1.0 min, 1.5 min, 2.0 min, 2.5 min, and 3.0 min. While maintaining other experimental conditions constant, the effects of plasma discharge power and cleaning times on cleaning efficacy are investigated by analyzing changes in the wettability, chemical composition, and chemical states of the stainless steel electrode surfaces before and after plasma cleaning.

### 2.2. Analysis Methods

#### 2.2.1. X-ray Photoelectron Spectroscopy (XPS)

X-ray photoelectron spectroscopy (XPS), a surface analysis technique known for its high sensitivity and resolution, is widely utilized in material surface analysis. XPS provides detailed information regarding the elemental composition, chemical states, valence states, and electronic structure within a depth of 1 to 10 nm from the material surface. Through XPS analysis, it is possible to accurately identify and quantify the types of elements present on the surface of cleaned stainless steel, revealing changes in the chemical states of various elements and potential compound formation. In this experiment, surface micro-element content of the samples was detected using an X-ray photoelectron spectrometer (XPS, PHI VersaProbe 4, London, UK). During the experiment, the XPS spectrum measurement utilized a pass energy of 224 eV and a step size of 0.8 eV, scanned twice, while the fine spectrum measurement utilized a pass energy of 69 eV and a step size of 0.125 eV, scanned six times. Calibration was performed using the C1s line at 284.50 eV, and Avantage software (Version 5.9931) was employed for high-resolution spectroscopic analysis of C and O elements.

#### 2.2.2. Water Contact Angle (WCA)

**Figure 3:**
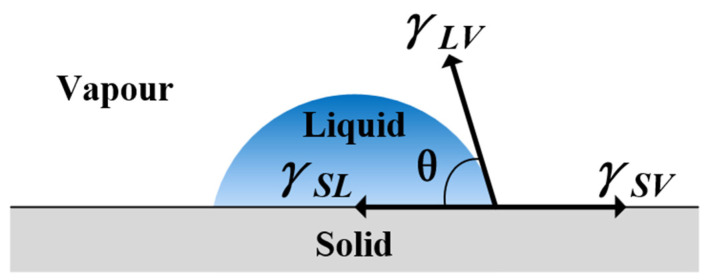
Diagram of water contact angle.

The water contact angle (WCA) is a crucial parameter for characterizing the wettability of material surfaces. It is defined as the angle formed by the tangent line at the point of contact between a liquid droplet and a solid surface, with respect to the surface of the electrode. When a liquid droplet comes into contact with a solid surface, there exists a three-phase intersection point of solid–liquid–gas at the surface of the solid. The angle (θ) between the tangent of the gas–liquid interface at the intersection point and the solid–liquid interface is defined as the contact angle of the droplet on the solid surface, as illustrated in Figure 3. The magnitude of the water contact angle reflects the wettability of the stainless steel electrode surface. In this experiment, the Theta Flex* (Biolin Scientific, Gothenburg, Sweden) optical contact angle measurement instrument was utilized to measure the contact angle between the sample surface and distilled water before and after plasma cleaning. When measuring the water contact angle, each water droplet has a volume of approximately 4 μL and is dropped onto the sample surface from a height of 1 mm.

## 3. Results and Discussion

### 3.1. Uniformity of Plasma Cleaning

**Figure 4:**
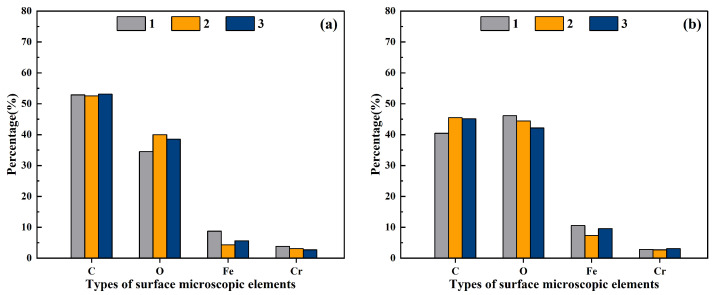
The proportion of chemical composition at three measuring points on the surface of stainless steel (**a**) power: 550 W; (**b**) power: 750 W.

**Figure 5:**
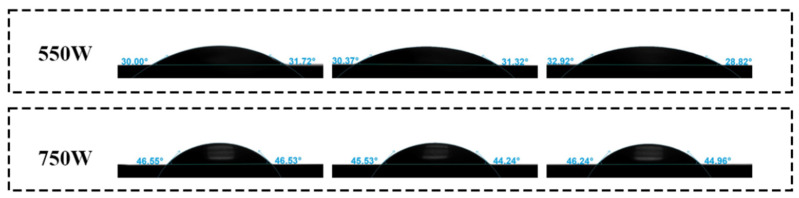
Water contact angles at different positions on the surface of stainless steel.

In plasma cleaning technology, the uniformity of the plasma jet plays a pivotal role in determining the efficacy of the cleaning process. However, the current array-type atmospheric pressure plasma jet suffers from a large gap problem, failing to achieve uniform cleaning of large-area material surfaces. This paper investigates the uniformity of the plasma jet generated by a rotating array-type plasma jet generator through water contact angle and XPS analysis (Figure 4 and Figure 5). Figure 4a,b show the results of surface microelement content detected by XPS technique at three measurement points on the stainless steel surface (as shown in Figure 2) after 1 min of plasma cleaning at discharge powers of 550 W and 750 W, respectively. When the discharge power was 550 W, the average carbon (C) content at different locations was 52.85%, with a standard deviation of 0.27%; oxygen (O) content averaged 37.69%, with a standard deviation of 2.84%; iron (Fe) content averaged 6.25%, with a standard deviation of 2.27%; chromium (Cr) content averaged 3.22%, with a standard deviation of 0.56%. When the discharge power was 750 W, the average carbon (C) content at different locations was 43.70%, with a standard deviation of 2.85%; oxygen (O) content averaged 44.25%, with a standard deviation of 1.99%; iron (Fe) content averaged 9.18%, with a standard deviation of 1.65%; chromium (Cr) content averaged 2.88%, with a standard deviation of 0.21%. The experimental results indicate that after plasma cleaning, the elemental content at three measurement points on the same sample is basically consistent.

Additionally, under the same experimental conditions, the water contact angle detected at any three positions on the stainless steel surface is shown in Figure 5. At a discharge power of 550 W, the water contact angles were 30.72°, 30.96°, and 30.87°, respectively. At a discharge power of 750 W, they were 46.23°, 45.07°, and 45.42°, respectively. These results indicate consistent water contact angle values across different locations on the sample surface. In summary, this paper proves that the rotating array-type plasma jet generator can produce large-area uniform plasma jets, ensuring consistent cleaning effects on the surface of stainless steel electrodes.

### 3.2. Chemical Composition Characteristics of the Stainless Steel Surface

**Figure 6:**
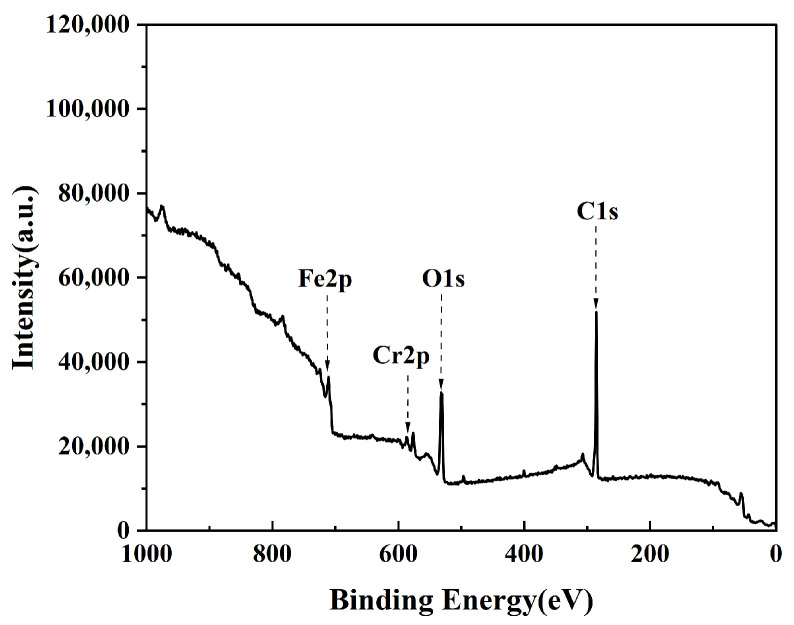
XPS Survey spectra of the untreated stainless steel electrode surface.

The chemical composition of the stainless steel surface undergoes significant changes after plasma cleaning. To further analyze the effect of plasma cleaning on the chemical properties of the stainless steel surface, this paper utilized XPS technology to characterize the surface of the samples before and after cleaning. The XPS Survey spectra of the stainless steel electrode surface before plasma treatment are presented in Figure 6, showing signals primarily attributed to C (1s), O (1s), Cr (2p), and Fe (2p), with relatively higher peaks for C and O.

**Figure 7:**
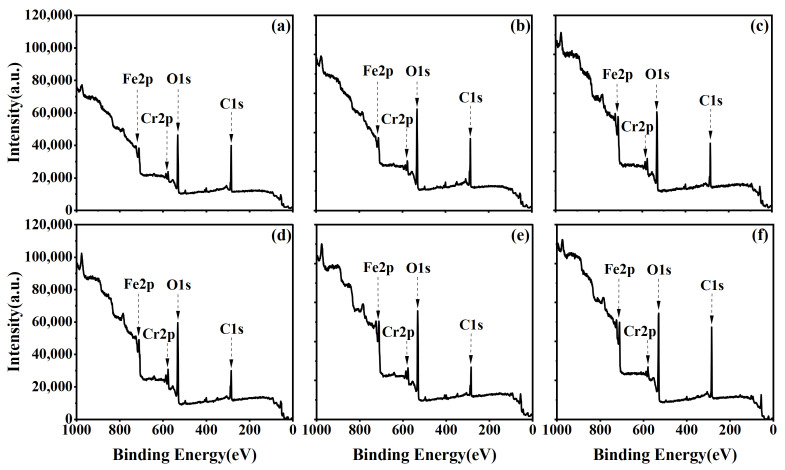
XPS Survey spectra of the stainless steel electrode surface under different cleaning times at a power of 550 W: (**a**) 0.5 min; (**b**) 1.0 min; (**c**) 1.5 min; (**d**) 2.0 min; (**e**) 2.5 min; (**f**) 3.0 min.

**Figure 8:**
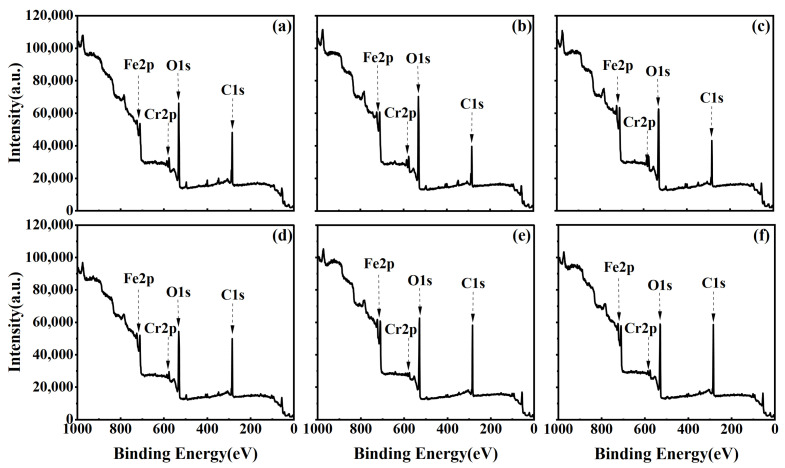
XPS Survey spectra of the stainless steel electrode surface under different cleaning times at a power of 750 W: (**a**) 0.5 min; (**b**) 1.0 min; (**c**) 1.5 min; (**d**) 2.0 min; (**e**) 2.5 min; (**f**) 3.0 min.

Figure 7a–f represent the XPS spectra corresponding to discharge powers ranging from 550 W, with cleaning times gradually increasing from 0.5 min to 3.0 min. Figure 8a–f illustrate the XPS Survey spectra corresponding to a cleaning power of 750 W. Analysis of XPS Survey spectra reveals significant changes in the peak intensities of C and O elements on the stainless steel surface after plasma cleaning. Under the experimental conditions of 550 W cleaning powers, an increase in plasma cleaning time leads to a decrease followed by an increase in C content. Conversely, the variation in O content exhibits an increase followed by a decrease. The overall trends in C and O element content under the 750 W cleaning condition are similar to those observed at 550 W.

**Figure 9:**
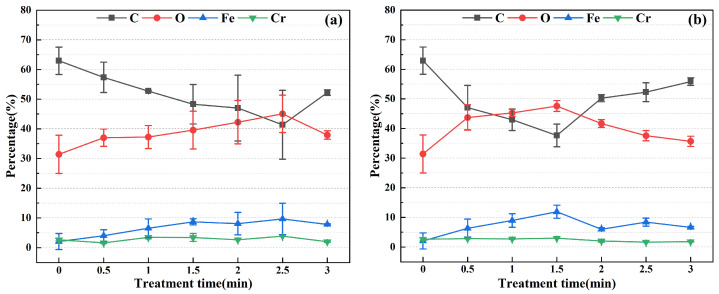
The change trend of the proportion of chemical composition on the surface of stainless steel under different cleaning parameters: (**a**) power: 550 W; (**b**) power: 750 W.

In this paper, the relative proportions of C, O, Fe, and Cr elements in the XPS Survey spectra were analyzed. The variations in relative proportions of surface elements on stainless steel electrodes under different cleaning parameters are illustrated in Figure 9.

As depicted in Figure 9a, the C content on the surface of the untreated stainless steel electrode is measured at 62.95%. Under a cleaning power of 550 W, an increasing time of plasma cleaning leads to a decreasing trend in C content. It reaches its nadir at 41.38% after 2.5 min of cleaning, representing a reduction of 21.57% compared to the untreated state. Beyond the 2.5 min, the C content begins to increase. In contrast, the trend in O content exhibits an initial increase followed by a decline, peaking at 45.08% after 2.5 min. Meanwhile, the Fe content initially increases with cleaning time before gradually stabilizing, while the variation in Cr content is negligible. At a cleaning power of 750 W (as shown in Figure 9b), the overall trends in C and O content mirror those observed at 550 W, although the minimum values for C and O content are reached at 1.5 min, registering at 37.68% and 47.61%, respectively, one minute earlier than at 550 W. The Fe content follows a similar pattern, increasing initially with cleaning time before decreasing, while the Cr content remains relatively unchanged. These results indicate that with increasing cleaning power, both the rate of change in elemental content and the time required to reach a critical state undergo alterations, which are related to the energy density of the plasma and reaction kinetics [28].

**Figure 10:**
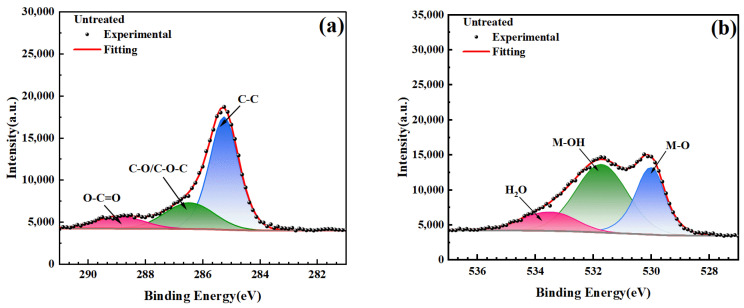
High-resolution XPS spectra fitting of untreated electrode surface signals: (**a**) C (1s); (**b**) O (1s).

To delve deeper into the changes in the microstructure and chemical states of stainless steel electrode surfaces before and after plasma treatment, this paper employed high-resolution XPS spectral fitting of C and O elements. In the high-resolution XPS analysis, the spectral characteristics of the C (1s) signal manifest as three distinct peaks, each corresponding to different chemical bonding environments, as shown in Figure 10a. The first peak, located at a binding energy of approximately 284.8 eV, represents electron transitions within C-C bonds, commonly observed in spectra of hydrocarbons. The second peak, located at a binding energy of approximately 286.0 eV, represents the chemical environment of the C-O/C-O-C, associated with the existence of oxygen functional groups such as alcohols and ethers. The third peak, located at a binding energy of approximately 288.5 eV, corresponds to characteristic transitions of carbonyl (O-C=O) functional groups, which are prevalent in ketones, aldehydes, as well as certain carboxylic acids and esters.

The high-resolution XPS spectra of the O (1s) signal also consists of three distinct components shown in Figure 10b. The first component represents oxygen atoms in metal oxides (M-O), such as Fe_2_O_3_ and Cr_2_O_3_, with a characteristic binding energy of 530.0 eV. The second component represents oxygen atoms in metal hydroxides (M-OH), such as Fe (OH)_3_ and Cr (OH)_3_, with a characteristic binding energy of 532.0 eV. The third component represents oxygen atoms in surface-adsorbed water molecules, with a characteristic binding energy of 533.0 eV.

**Figure 11:**
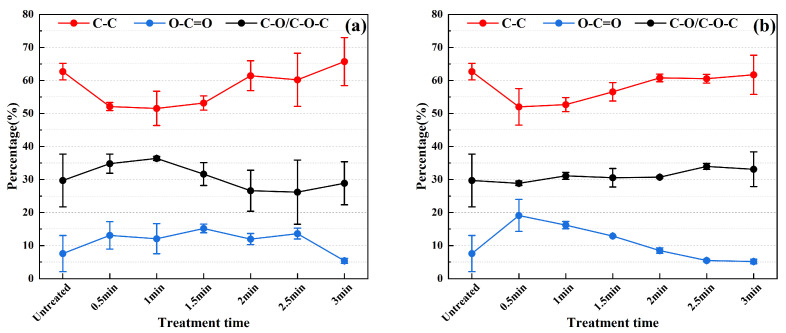
The change trend of the chemical state of C element under different cleaning parameters: (**a**) power: 550 W; (**b**) power: 750 W.

**Figure 12:**
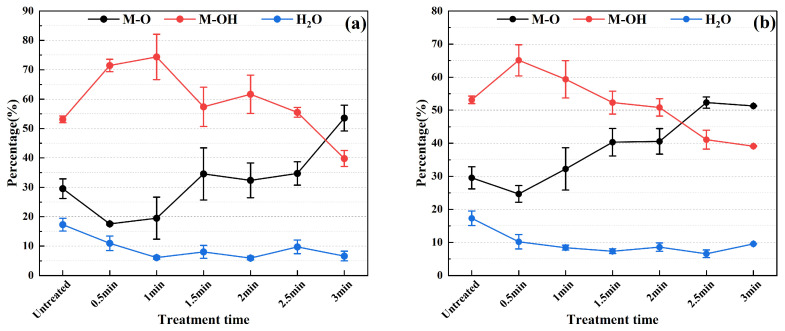
The change trend of the chemical state of O element under different cleaning parameters: (**a**) power: 550 W; (**b**) power: 750 W.

The relative proportions of C-C, O-C=O, C-O/C-O-C, M-O, M-OH, and H_2_O were calculated based on the high-resolution XPS spectra data of C and O elements as shown in Figure 11 and Figure 12. Figure 11 indicates that, under discharge powers of 550 W and 750 W, the relative content of C-C initially decreases before subsequently increasing with cleaning time, while the relative content of O-C=O experiences an initial increase followed by a decrease. Furthermore, the relative content of C-O/C-O-C also shows some fluctuations.

Based on the XPS analysis results of the chemical states of O elements shown in Figure 12, it can be observed that during plasma cleaning, the relative content of hydroxides on the stainless steel electrode surface exhibits an initial increase followed by a decrease, while the relative content of oxides shows an initial decrease followed by a gradual increase. Furthermore, the relative content of surface-adsorbed water continuously decreases throughout the entire cleaning period, decreasing from 17.31% to 5.9% at 550 W and from 17.31% to 6.6% at 750 W. The specific mechanism underlying the change in the chemical states of C and O elements on the stainless steel surface after plasma cleaning will be further analyzed in the next section.

### 3.3. Evolution of Surface Microscopic Elemental Content and Chemical State

**Figure 13:**
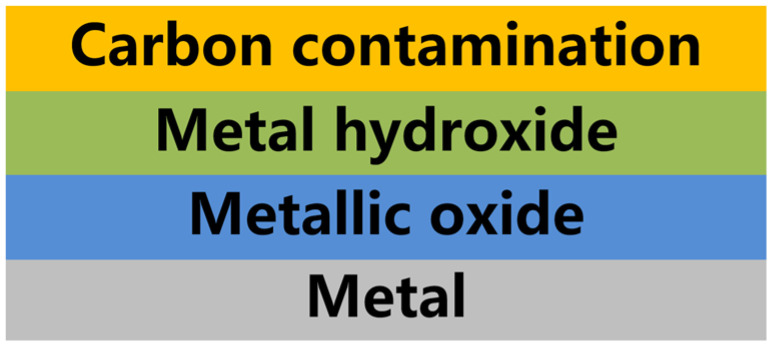
Simplified model of surface pollutants on stainless steel electrodes exposed to atmospheric environment.

The classical model of surface pollutants on stainless steel electrodes is shown in Figure 13 [29,30]. This model comprises the following layers from top to bottom: a carbon contamination layer (C), a layer of metal hydroxides (M-OH), a layer of metal oxides (M-O), and finally, the metal layer (Metal). Since the contamination layer on stainless steel electrode surfaces is typically around 3–15 µm thick, in most cases, these stages often overlap and occur concurrently.

**Figure 14:**
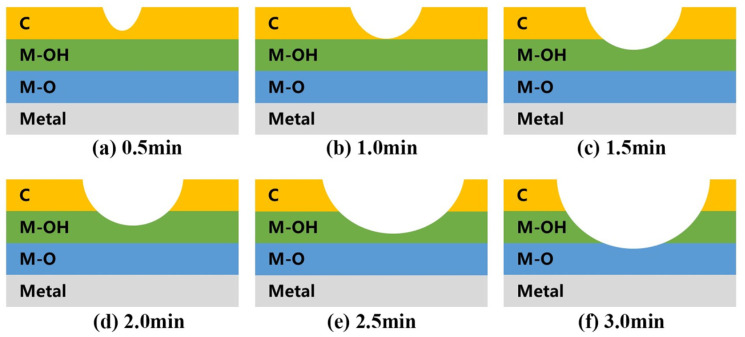
Plasma cleaning model of the stainless steel electrode surface at different cleaning times at a power of 550 W.

**Figure 15:**
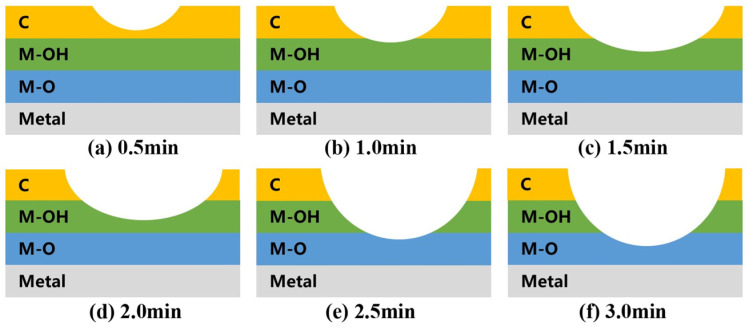
Plasma cleaning model of the stainless steel electrode surface at different cleaning times at a power of 750 W.

Based on the proportions of surface elements obtained from the XPS spectra, along with the chemical states of C and O elements fitted from high-resolution XPS spectra, Table 1 presents the cleaning ranges under different cleaning parameters. Simultaneously, stainless steel electrode surface cleaning models corresponding to different experimental conditions are illustrated in Figure 14 and Figure 15.

The chemical composition and states of the surface of stainless steel electrodes are crucial factors determining their performance. Among these factors, the content and distribution of C and O elements on the surface significantly influence the chemical properties of stainless steel materials. Therefore, this paper integrates the plasma cleaning ranges and models under various cleaning conditions mentioned above to conduct a detailed analysis of the changes in the relative content and chemical states of C and O elements in the XPS analysis results. The results in Figure 9 indicate that under discharge powers of 550 W and 750 W, as cleaning time increases, the relative content of C elements initially decreases before increasing, while the change in O elements exhibits the opposite trend, with inflection points occurring at cleaning times of 2.5 min and 1.5 min, respectively.

The variation in the relative content of C can be attributed to the following factors: Initially, the stainless steel electrode surface may already harbor contaminants such as oil, dust, and oxide layers. During the initial stages of plasma cleaning, high-energy particles (such as electrons and ions) effectively remove these contaminants, leading to a decrease in the proportion of C elements. As plasma cleaning progresses, carbon-based contaminants on the stainless steel surface are removed, while surface activity is enhanced, promoting reactions between carbon dioxide (CO_2_) or other carbon-containing gases in the air and the surface, forming new carbon-containing compounds, and thereby increasing the relative content of C elements.

Concurrently, the variation in O elements is due to the removal of the top layer of carbon contaminants on the electrode surface by plasma cleaning, exposing the surface oxide layer. Additionally, oxygen atoms in the plasma react with iron atoms to form iron oxides (such as Fe_2_O_3_ or Fe_3_O_4_), thereby increasing the relative content of O elements on the surface. With prolonged cleaning time, the high temperature and high-energy particles in the plasma promote the decomposition or evaporation of oxides. Moreover, continuous plasma cleaning results in the reorganization of the surface structure, causing originally exposed oxides to be re-embedded or covered again. Furthermore, the electrode surface gradually reaches an oxide saturation state, slowing down the oxidation rate, and ultimately resulting in a decrease in the relative content of O elements.

Based on the high-resolution XPS spectral data of the C (1s) signal (Figure 11), at a discharge power of 550 W, the observed initial increase followed by a subsequent decrease in the relative ratio of C-O/C-O-C suggests a gradual enhancement of oxidation. As cleaning time progresses, the C-O/C-O-C ratio initially increases due to the strengthening of oxidation. However, with prolonged cleaning, C-O/C-O-C further oxidizes to form O-C=O, leading to a reduction in the relative ratio of C-O/C-O-C. Conversely, at a discharge power of 750 W, where oxidation is more pronounced, the conversion of C-O/C-O-C to O-C=O occurs rapidly, resulting in less perceptible changes in the proportion of C-O/C-O-C throughout the cleaning process. The initial decline in the proportion of C-C is attributed to the removal of carbon-containing contaminants, while the subsequent rise primarily stems from the formation of new carbon compounds and the reduction in the proportions of C-O/C-O-C and O-C=O.

According to the high-resolution XPS spectral data of the O (1s) signal (Figure 12), in the initial stages of plasma cleaning (corresponding to 1 min cleaning at 550 W power and 0.5 min cleaning at 750 W power), the heightened surface activity of stainless steel promotes the reaction between water molecules in the air and the surface, leading to the formation of M-OH and, consequently, an increase in the relative proportion of M-OH alongside a decrease in the relative proportion of M-O. As cleaning time progresses, the cleaning action gradually extends to include M-OH, which undergoes further decomposition or oxidation to yield more stable M-O, resulting in a decline in the relative proportion of M-OH and a simultaneous increase in the relative proportion of M-O. When cleaning time reaches 3 min at 550 W power or 2.5 min at 750 W power, the cleaning range begins to extend to the M-O layer, at which point the relative proportion of M-O surpasses that of M-OH. Additionally, owing to the thermal effects of the plasma and the bombardment of high-energy particles, the adsorbed H_2_O on the surface may gradually evaporate or desorb, leading to a continuous decrease in the relative proportion of H_2_O. These observations reflect the profound influence of plasma cleaning on the surface chemical composition of stainless steel and underscore the critical role of cleaning parameters in governing surface chemical states.

### 3.4. Evolution of Surface Microscopic Elemental Content and Chemical State

**Figure 16:**
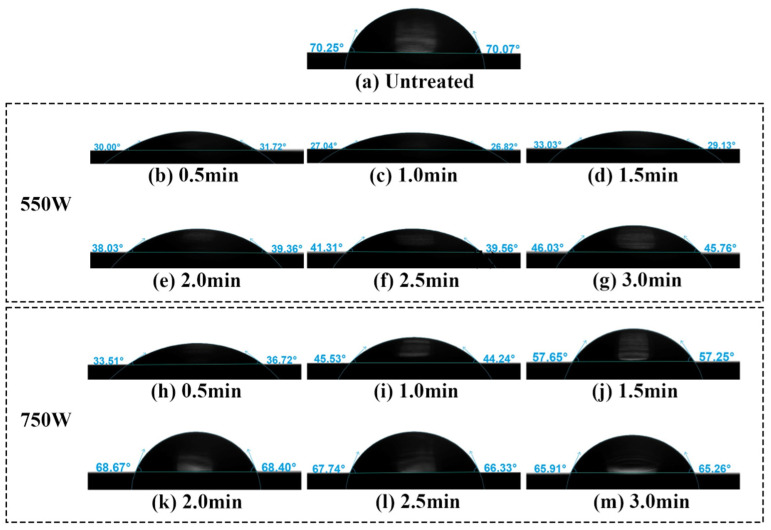
Water contact angle of the stainless steel electrode surface before and after plasma cleaning: (**a**) untreated; (**b**–**g**) power: 550 W; (**h**–**m**) power: 750 W.

**Figure 17:**
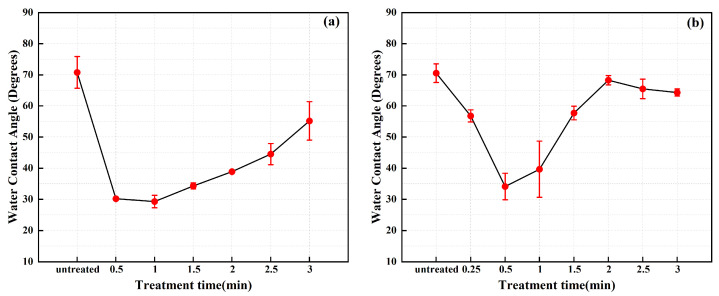
The change trend of water contact angle on the surface of stainless steel electrode before and after plasma cleaning (averages ± standard deviations): (**a**) power: 550 W; (**b**) power: 750 W.

Figure 16 illustrates the variation in water contact angles on the surface of stainless steel electrodes under different plasma cleaning parameters. Figure 16a represents the initial state of the water contact angle on the untreated electrode surface. Figure 16b–g respectively present the trend of water contact angle variation on the electrode surface as cleaning time gradually increases from 0.5 min to 3.0 min at a cleaning power of 550 W. Figure 16h–m present the trend of water contact angle variation on the stainless steel electrode surface at different cleaning times when the cleaning power is increased to 750 W. To provide a more intuitive analysis of the trend in water contact angles, the data from Figure 16 are plotted as line graphs in Figure 17.

From Figure 17, it can be observed that at a discharge power of 550 W, the water contact angle on the electrode surface decreases significantly as cleaning time increases, dropping from approximately 70.76° to around 29.31°, reaching its minimum value at 1 min, and then gradually increases. At a discharge power of 750 W, the water contact angle reaches its minimum value of approximately 34.12° after 0.5 min of cleaning, gradually stabilizing at around 65° after 2 min. Overall, with the extension of plasma cleaning time, the water contact angle on the stainless steel electrode surface exhibits a trend of initially decreasing followed by increasing.

For discharge powers of 550 W and 750 W, the plasma cleaning times required to reach the minimum water contact angle on the electrode surface are 1 min and 0.5 min, respectively. Increasing the power enhances the energy density in the plasma, leading to increased activity of ions and active species, thereby accelerating the cleaning process. As cleaning time extends, the water contact angle on the stainless steel electrode surface shows a trend of initially decreasing followed by increasing, indicative of an enhancement succeeded by a downturn in the wettability of the stainless steel electrode surface.

### 3.5. Mechanism of Surface Wettability Improvement

The active particles in the plasma jet can influence the surface properties of stainless steel, particularly its wettability. When pollutants cover the surface of stainless steel electrodes, they create a significant energy barrier, hindering liquid penetration onto the surface. Plasma cleaning effectively removes these pollutants, thereby improving the wettability of the stainless steel electrode surface. Additionally, during the cleaning process, high-energy particles in the plasma (such as electrons, ions, and radicals) interact with the stainless steel surface, causing chemical changes on the material surface. These chemical changes can modify the energy state and chemical composition of the stainless steel surface, making it more prone to contact with liquids. Consequently, stainless steel electrode surfaces treated with plasma cleaning often exhibit superior wettability compared to untreated surfaces.

The water contact angle measurements presented in this paper indicate a non-monotonic variation of the water contact angle on the surface of stainless steel electrodes with increasing cleaning time. Specifically, the water contact angle initially decreases, followed by a gradual increase. This phenomenon reflects a trend of enhanced wettability followed by attenuation on the surface of stainless steel electrodes. The change in wettability is attributed to the interaction between high-energy particles in the plasma and surface contaminants on the stainless steel surface, such as oils, dust, and oxides, during the early stages of cleaning. This interaction promotes the exposure or formation of hydrophilic functional groups (such as -OH and O-C=O) on the surface, thereby reducing the water contact angle. As cleaning progresses, the degree of surface activation increases, leading to the formation of new chemical bonds or structures, which may contain more hydrophilic functional groups, further facilitating a decrease in the water contact angle. However, prolonged cleaning time can result in excessive surface etching, altering the surface’s microstructure and causing changes in wettability, ultimately resulting in an increase in the water contact angle.

Existing studies have demonstrated that the changes in surface wettability of electrodes after plasma cleaning are primarily influenced by hydroxyl (-OH) and carbonyl (O-C=O) hydrophilic functional groups [31,32]. The higher the content of hydroxyl (-OH) on the material surface, the better the wettability. The specific mechanism by which hydroxyl (-OH) affects wettability is as follows: the hydroxyl group exhibits strong polarity, enabling it to form hydrogen bonds with water molecules. This hydrogen bonding interaction facilitates the adsorption of water molecules onto the material surface, thereby enhancing the surface’s wettability; the presence of hydroxyl groups enhances the material surface’s ability to adsorb water molecules, making it easier for water molecules to adsorb and spread on the surface, thereby reducing the contact angle of water [33,34].

In the plasma cleaning process described in this paper, -OH groups mainly originate from the interaction between water molecules and surface metal atoms, including hydrolysis reactions and oxidation reactions. The corresponding chemical reactions can be represented as follows:

(1) $$H_{2}O\;\to \;H\cdot +\cdot \mathrm{OH}$$

(2) $$\mathrm{Fe}+O_{2}\;\to \;\mathrm{FeO}$$

(3) $$\mathrm{FeO}+H_{2}O\;\to \;{\mathrm{Fe}(\mathrm{OH})}_{2}$$

(4) $${2\mathrm{Fe}(\mathrm{OH})}_{2}+O_{2}\;\to \;2\mathrm{FeOOH}$$

(5) $$\mathrm{Cr}+O_{2}\;\to \;{\mathrm{CrO}}_{2}$$

(6) $${\mathrm{CrO}}_{2}+H_{2}O\;\to \;{\mathrm{Cr}(\mathrm{OH})}_{2}$$

The O-C=O mainly originates from the reaction between high-energy oxygen atoms or oxygen molecules in the plasma and carbon atoms or carbon compounds on the surface of stainless steel. The corresponding chemical reactions can be represented as follows:

(7) C+O· → CO·

(8) $$\mathrm{CO}\cdot +\;O_{2}\;\to \;{\mathrm{CO}}_{3}$$

(9) $$\mathrm{FeO}+{\mathrm{CO}}_{2}\;\to \;{\mathrm{FeCO}}_{3}$$

(10) $${\mathrm{FeCO}}_{3}+H_{2}O\;\to \;{\mathrm{Fe}({\mathrm{CO}}_{3})}_{2}\;+\;H^{+}$$

The change trend of M-OH and O-C=O depicted in Figure 11 and Figure 12 shows that with increasing cleaning time, the relative content of -OH and O-C=O both initially increases and then decreases. It is noteworthy that the contribution of O-C=O to hydrophilicity is significantly weaker compared to -OH [35]. Consequently, the wettability of the stainless steel electrode surface exhibits a trend of initial enhancement followed by reduction, consistent with the trend of contact angle with water decreasing initially and then increasing.

### 3.6. Time Efficiency Characteristics of the Plasma Cleaning

In practical applications, the effect of the sample surface after plasma cleaning is time efficiency, gradually leading it back to its original state over time. This phenomenon occurs as the material surface may reabsorb environmental contaminants or undergo chemical changes. Therefore, it is necessary to assess the stability and durability of the stainless steel electrode surface performance after cleaning through systematic time efficiency tests.

**Figure 18:**
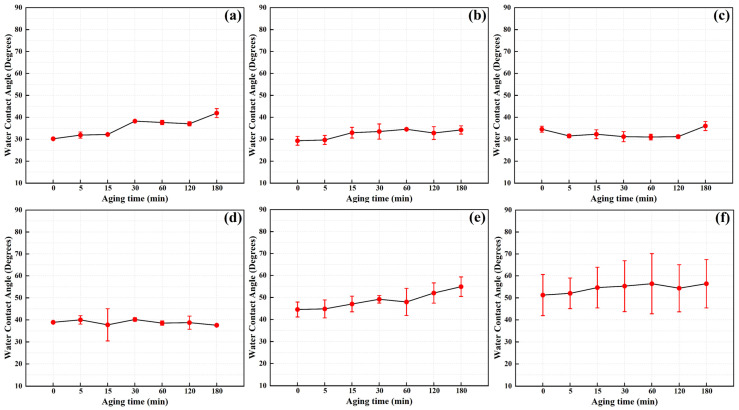
Time efficiency characteristics of plasma cleaning efficiency under different cleaning times at a power of 550 W (averages ± standard deviations): (**a**) 0.5 min; (**b**) 1.0 min; (**c**) 1.5 min; (**d**) 2.0 min; (**e**) 2.5 min; (**f**) 3.0 min.

**Figure 19:**
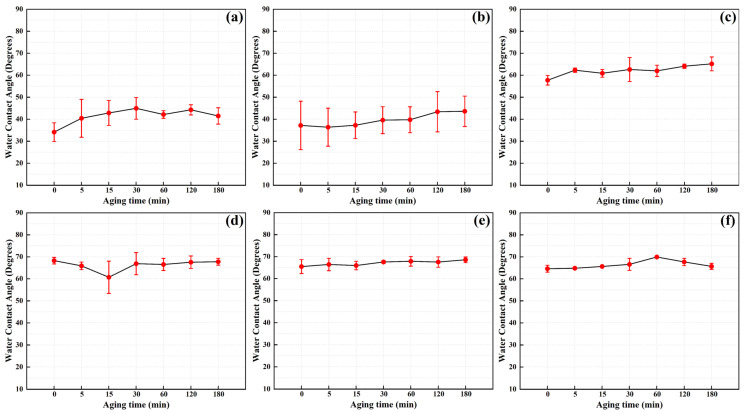
Time efficiency characteristics of plasma cleaning efficiency under different cleaning times at a power of 750 W (averages ± standard deviations): (**a**) 0.5 min; (**b**) 1.0 min; (**c**) 1.5 min; (**d**) 2.0 min; (**e**) 2.5 min; (**f**) 3.0 min.

Under different discharge powers and cleaning times, cleaned electrodes were exposed to atmospheric conditions for 3 h to monitor the trend of water contact angle changes on the electrode surface during exposure, as illustrated in Figure 18 and Figure 19. It is evident that the samples treated with plasma cleaning maintain their effectiveness within the 180 min exposure period. Specifically, samples treated for 1 min or 1.5 min at a discharge power of 550 W exhibit good wettability and maintain stability during the observation period. However, when the discharge power is increased to 750 W, samples treated for 0.5 and 1 min demonstrate the best wettability and time efficiency characteristics.

**Figure 20:**
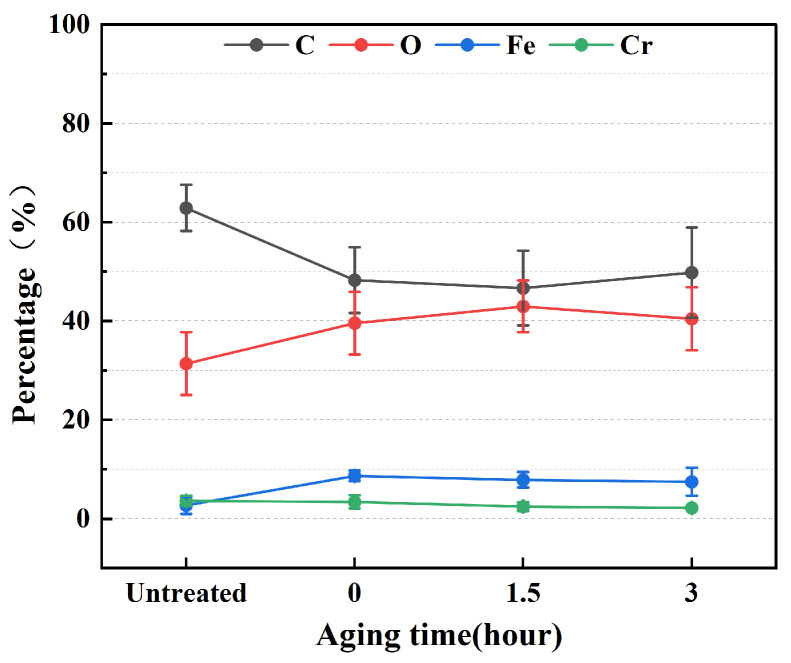
XPS detection results of the stainless steel electrode surface exposed to different times in the atmospheric environment.

In addition, under the experimental conditions of a discharge power of 550 W, working pressure of 150 kPa, and cleaning time of 1.5 min, the stainless steel electrode surface was cleaned using plasma cleaning. Subsequently, the samples were exposed to atmospheric conditions, and XPS analysis was conducted at 0 h, 1.5 h, and 3 h after cleaning to quantitatively assess the chemical composition changes on the stainless steel surface, as shown in Figure 20. The results indicate that after plasma cleaning, the C content on the stainless steel electrode surface decreased significantly by approximately 14%, while the O content increased by approximately 13%. The Fe content increased slightly by around 5%, with no significant change observed in the Cr content. Furthermore, the trends in elemental content remained stable over the 3 h period, consistent with the results of time efficiency tests conducted for the water contact angle on the stainless steel electrode surface. These results show that the chemical modification effect of plasma cleaning on the surface of stainless steel has a certain stability, and the effect is less susceptible to atmospheric influences in the short term.

## 4. Conclusions

This study utilized a rotating array plasma jet generator to produce a large-area uniform plasma jet for cleaning the surface of stainless steel electrodes. The wettability, chemical composition, and chemical states of the electrode surface were characterized by using water contact angle measurements and X-ray photoelectron spectroscopy (XPS). The cleaning effects under different discharge powers and cleaning times were analyzed, and the cleaning mechanism of contaminants on the stainless steel electrode surface was investigated. The main conclusions are as follows:

1. Plasma cleaning significantly improved the wettability of the stainless steel electrode surface, as evidenced by a decrease in the water contact angle from 70.76° to 29.31°. Additionally, the surface carbon content decreasing from 62.95% to 37.68%, indicating effective removal of carbon contaminants. At discharge powers of 550 W and 750 W, the cleaning times required to achieve the lowest carbon contamination were 2.5 min and 1.5 min, respectively. Regarding wettability and ageing characteristics of the samples, the optimal cleaning condition was observed at a discharge power of 550 W with a cleaning time of 1.0 min.
2. The XPS analysis results of the electrode surface, combined with the water contact angle data, demonstrate a highly consistent trend between the content of -OH and O-C=O functional groups and the wettability of the electrode surface. This indicates that -OH and O-C=O are the primary functional groups influencing the wettability of the electrode surface.
3. The results of time efficiency characteristics tests indicate that within 3 h of exposure to ambient air following plasma cleaning, there were no significant changes observed in the water contact angle and surface elemental content. This suggests a certain level of stability in the cleaning effectiveness of plasma cleaning on the stainless steel surface.

## Figures and Tables

**Table 1:** Plasma cleaning range under different cleaning parameters.

Power	Cleaning Time	Cleaning Scope	Power	Cleaning Time	Cleaning Scope
550 W	0.5 min	C	750 W	0.5 min	C
	1.0 min	C		1.0 min	C, M-OH
	1.5 min	C, M-OH		1.5 min	C, M-OH
	2.0 min	C, M-OH		2.0 min	M-OH
	2.5 min	C, M-OH		2.5 min	M-OH, M-O
	3.0 min	M-OH, M-O		3.0 min	M-OH, M-O

## Data Availability

Data are contained within the article.
